# Endoscopic endonasal approach for pituitary neuroendocrine tumor with septal mucosa incision tailored to tumor extension intending unilateral septal mucosa preservation

**DOI:** 10.1038/s41598-024-84334-y

**Published:** 2025-01-09

**Authors:** Hiroyoshi Kino, Hiroyoshi Akutsu, Shuho Tanaka, Takuma Hara, Yusuke Morinaga, Hidetaka Miyamoto, Rieko Ii, Koutarou Osawa, Eiichi Ishikawa

**Affiliations:** 1 Department of Neurosurgery, Faculty of Medicine, University of Tsukuba, Ibaraki, Japan; 2 Department of Neurosurgery, Dokkyo Medical University, 880 Kitakobayashi, Mibu, Tochigi 321-0293 Japan; 3 Department of Otolaryngology, Faculty of Medicine, University of Tsukuba, Ibaraki, Japan

**Keywords:** Pituitary neuroendocrine tumor, Endoscopic endonasal surgery, Paraseptal approach, Killian incision, rescue flap incision, Olfactory system, Cancer, Endocrine system and metabolic diseases

## Abstract

Currently, the direct endonasal approach is widely used in endoscopic endonasal surgery (EES) for pituitary neuroendocrine tumor. However, a large posterior septal perforation is inevitable. We routinely utilize a modified para/transseptal approach using the combination of a Killian and a contralateral rescue flap incision (PTSA with K-R incision). Especially, rescue flap incision is used on ipsilateral side of the tumor extension to facilitate lateral expansion of the anterior sphenoidotomy and tumor resection. One hundred eighty-nine patients underwent EES using PTSA with K-R incision in Tsukuba university hospital. Gross total resection (GTR) was achieved in 146 (77.2%) patients. Even in cases of significant lateral tumor extension (Knosp 3 or 4), GTR was achieved in 56 of 90 (62.2%) patients. Among the 168 patients with available rhinological follow-up data, postoperative anterior and posterior septal perforation occurred in six (3.6%) and 18 (10.7%) patients, respectively. Surgical intervention was required in one (0.6%) patient with anterior septal perforation. Thus, PTSA with K-R incision was effective in preserving the nasal septal mucosa on one side and gaining a sufficient surgical corridor with lateral expansion of the anterior sphenoidotomy on the other side. Our strategy may be preferable to improve maneuverability of instruments during tumor resection.

**Supplementary Information:**

The online version contains supplementary material available at 10.1038/s41598-024-84334-y.

## Introduction

**Fig. 1:**
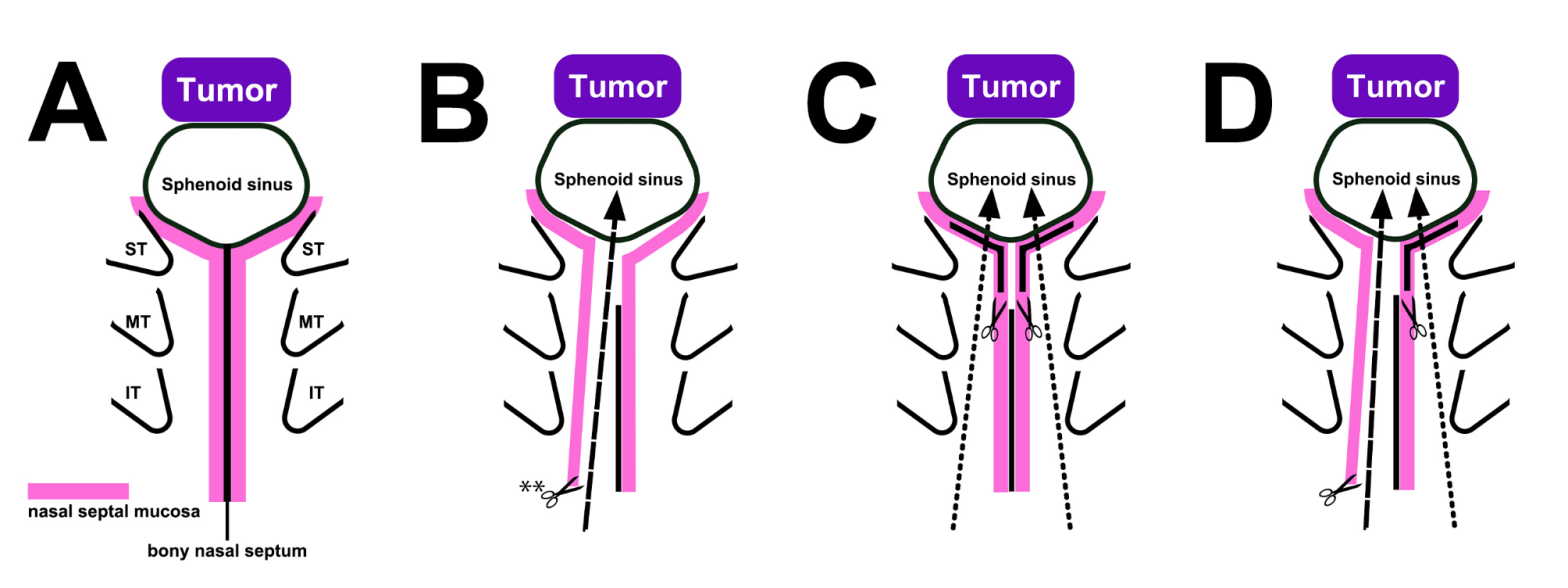
Several methods of approaching the sphenoid sinus. (**A**) Schematic diagram of the nasal septal mucosa, sphenoid sinus, and nasal turbinate. ST: superior turbinate. MT: middle turbinate. IT: inferior turbinate. (**B**) Schematic diagram of the PTSA method. The mucosa of the nasal septum is dissected from the front, and reaching the sphenoid sinus is possible without disturbing the nasal turbinate. **Vertical incision of the nasal septal mucosa. (**C**) Schematic diagram of the direct endonasal approach. In this approach, the nasal septal mucosa is incised laterally near the sphenoid sinus to directly approach the inside of the sphenoid sinus. (**D**) Schematic diagram of PTSA with K-R incision. PTSA is used on one side and Rescue flap incision used on the other side.

In the endoscopic endonasal approach (EEA) for pituitary neuroendocrine tumors (PitNETs), several methods of approaching the sphenoid sinus have been reported^1–11^. For sufficient opening of the anterior wall of the sphenoid sinus, which is the key procedure of EES, the nasal septal bone and mucosa (Fig. 1A) should be manipulated. These methods are categorized as para/transseptal (Fig. 1B) and direct endonasal approaches (direct approach) (Fig. 1C). The para/transseptal approach (PTSA) using the ipsilateral or bilateral vertical incision is known as the Killian incision (Fig. 1B)^12^. Using this technique, unilateral septal mucosa and nasal turbinates are completely preserved; however, submucosal dissection of the nasal septal mucosa is technically demanding and may require longer time. Additionally, the preserved nasal septal mucosa limits lateral exposure of the sphenoid sinus anterior wall^3^. Conversely, the direct approach using bilateral Rescue flap incision (Fig. 1C) is quick and simple; therefore, it is widely used in pituitary surgeries^13–15^. In this approach, an anterior sphenoidotomy can be easily laterally expanded. However, postoperative large posterior septal perforation is inevitable; additionally, bilateral septal mucosa and nasal turbinates are possibly damaged by moving surgical instruments through the nasal cavity^16^. This can lead to empty nose syndrome or adhesion of the nasal cavity, causing subsequent nasal obstruction or pain^16,17^.

To preserve the nasal septal mucosa and nasal turbinates and enable lateral expansion of the anterior sphenoidotomy to facilitate tumor resection, we routinely utilize modified PTSA for PitNETs using the combination of a Killian incision and a contralateral rescue flap incision (PTSA with K-R incision; Fig. 1D); in doing so, we firstly select the septal mucosal incision side according to the direction of the tumor extension. We operate not only on PitNETs but also other skull base tumors as craniopharyngiomas, meningiomas, and chordomas using the same approach. In this study, we describe the technical details and clinical outcomes of this method with special reference to postoperative septal perforation evaluated by fiberscopic observation.

## Materials and methods

This was a retrospective analysis of 202 consecutive patients who underwent EEA for PitNET (n = 137 with non-functioning PitNET and n = 65 with functioning PitNET) from January 2016 to December 2020 in Tsukuba university hospital. PTSA with K-R incision was used in 189 (93.1%) of the 202 patients who underwent EEA for PitNETs. Among the 13 patients who did not undergo PTSA with the K-R incision, six had undergone a prior surgery using the direct approach with bilateral rescue flap incision in other hospitals. Therefore, the same direct approach with bilateral rescue flap incision was used in the surgery performed at our hospital. These 6 patients were also analyzed as control group of direct approach. The remaining seven patients had an NSF prepared at the beginning of the surgery because an intraoperative high-flow CSF leak was expected; therefore, these patients were excluded in this study.

All surgeries were performed by an interdisciplinary collaborative team of neurosurgeons and otolaryngologists. All patients were evaluated using magnetic resonance imaging (MRI) preoperatively and postoperatively at 1 week, 3 months, and then every year thereafter to detect residual or recurrent tumors. Lateral tumor extension was categorized based on Knosp gradings for preoperative MRI. Knosp grades were defined as follows: 0 and 1: no lateral extension; 2: mild lateral extension; 3 and 4: significant lateral extension^18^. The side of lateral tumor extension was categorized as left, right, bilateral, or none using Knosp grading. Computed tomography (CT) was obtained preoperatively and at approximately 6 months postoperatively to evaluate the nasal structure including septum deviation, sinusitis, or mucocele. The presence of a nasal septum deviation was determined on the basis of preoperative CT findings and the side of septum deviation was categorized as left, right, or none. The degree of septum deviation was evaluated by measuring the distance between the midline to the most prominently deviated point.

**Fig. 2:**
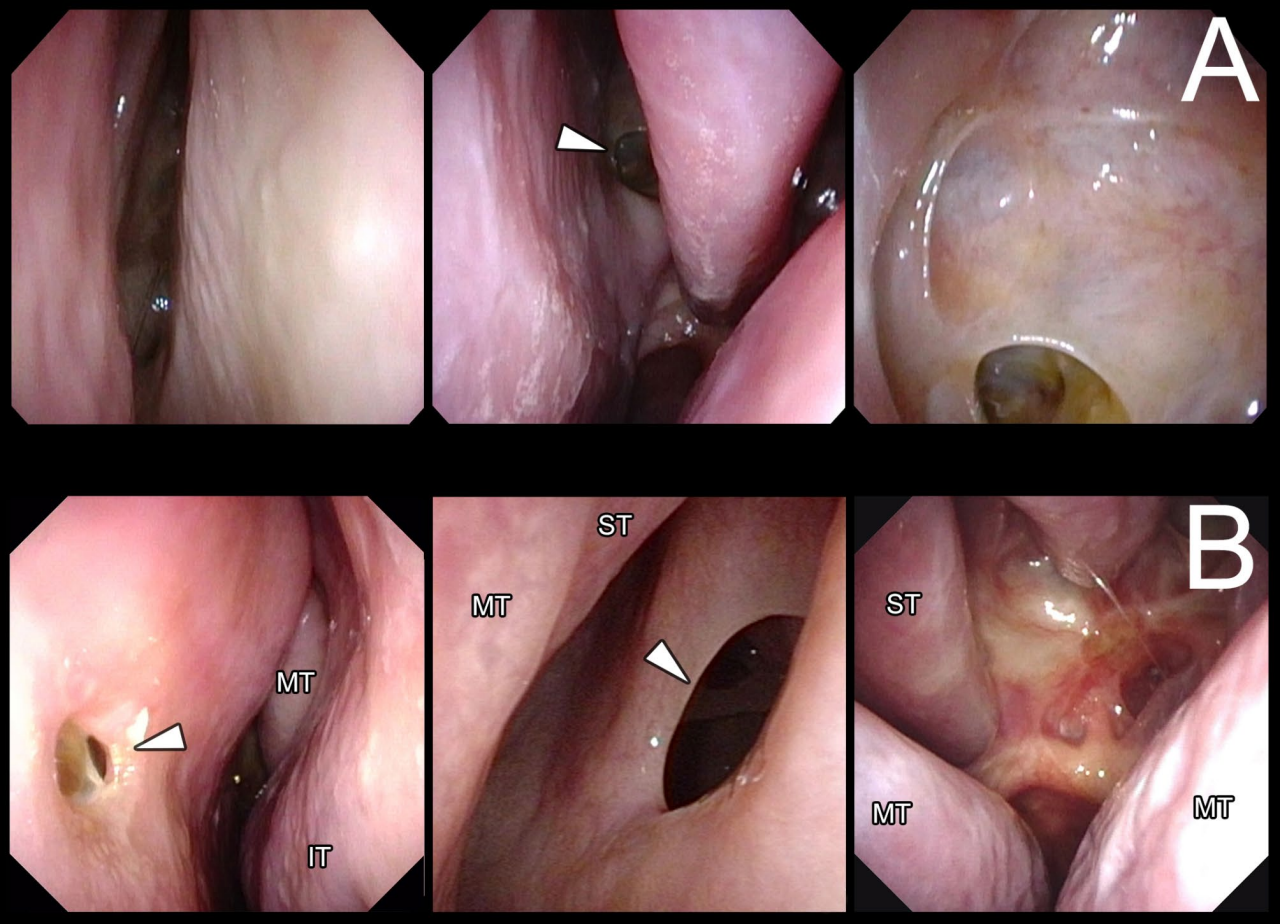
Postoperative fiberscopic view of the bilateral nasal septal mucosa and sphenoid sinus. (**A**) Condition of a nasal septal mucosa and sphenoid sinus after the para/transseptal approach using the combination of the Killian incision and contralateral rescue flap incision (PTSA with K-R incision). Left, the nasal septal mucosa is completely preserved on the right side of the Killian incision. Middle, most of the nasal septal mucosa is preserved, with the exception of the area around the sphenoid ostium on the left side of the rescue flap incision. Right, condition of the operative site in the sphenoid sinus can be observed through the mucosal defect around the sphenoid ostium on the left side of the rescue flap incision. (**B**) Nasal septal perforation after PTSA with the K-R incision (left and middle) and after the direct endonasal approach (right). Left, anterior septal perforation. Middle, posterior septal perforation. Right, large posterior septal perforation after the direct endonasal approach using bilateral rescue flap incisions. Abbreviations: IT, inferior turbinate; MT, middle turbinate; ST, superior turbinate.

The extent of tumor resection was classified as gross total resection (GTR; 100%), subtotal resection (STR; ≥ 90%), and partial resection (PR; < 90%). For all patients, nasal cleaning and direct observation of the nasal septal mucosa and sphenoid sinus were conducted using a flexible endoscope by otolaryngologists at every postoperative visit for at least 3 months. Rhinological follow-up continued but ceased at approximately 6 months if there was no problem. On the electrical charts, pictures and/or movies taken with a fiberscope were attached and the presence of a septal perforation or sinusitis was noted (Fig. 2A,B). A nasal septal perforation was classified as anterior or posterior based on the location relative to the anterior margin of the middle turbinate (Fig. 2B). For all cases with nasal septal perforation, patients were questioned by telephone with a structured questionnaire regarding four complications: whether they experienced complicated epistaxis, nasal obstruction, increased nasal crusting, and nasal whistling postoperatively. An additional revision surgery for septal perforation was considered if patients experienced severe complications. To compare septal perforation using PTSA with K-R incision versus the direct approach, all patients were observed to identify septal perforation and questioned regarding the above nasal symptoms. Patients were defined as having postoperative sinusitis if they received antibiotic treatment or surgery. Postoperative epistaxis was defined as the need for any hemostatic procedures by an otolaryngologist, including stuffing gauze or coagulation. A new postoperative mucocele was identified if the postoperative CT or MRI at 3–6 months showed marked fluid collection in the paranasal sinus that was not associated with sinusitis. The factors associated with a postoperative anterior or posterior septal perforation were analyzed; these were stratified by sex, age, significant lateral tumor extension (Knosp 3 or 4), maximum tumor diameter, achieving GTR, the existence of nasal septum deviation, discrepancy between the side of lateral tumor extension and nasal septum deviation, degree of nasal septum deviation, and a history of previous pituitary surgery, and acromegaly.

### Surgical technique for PTSA with K-R incision (see supplementary videos 1, 2)

The side indicated as the lateral tumor extension on preoperative MRI was used for rescue flap incision. Concurrently, considering preservation of the nasal septal mucosa in the presence of septal deviation, the side of the Killian incision was selected as being the same side as the wider concave side of the nasal septum, where a paraseptal submucosal dissection was easier. However, the tumor extension to the septum deviation was given priority for mucosal incision; if there was no lateral tumor extension, the selection of the mucosal incision depended on the side of the nasal septum deviation.

**Fig. 3:**
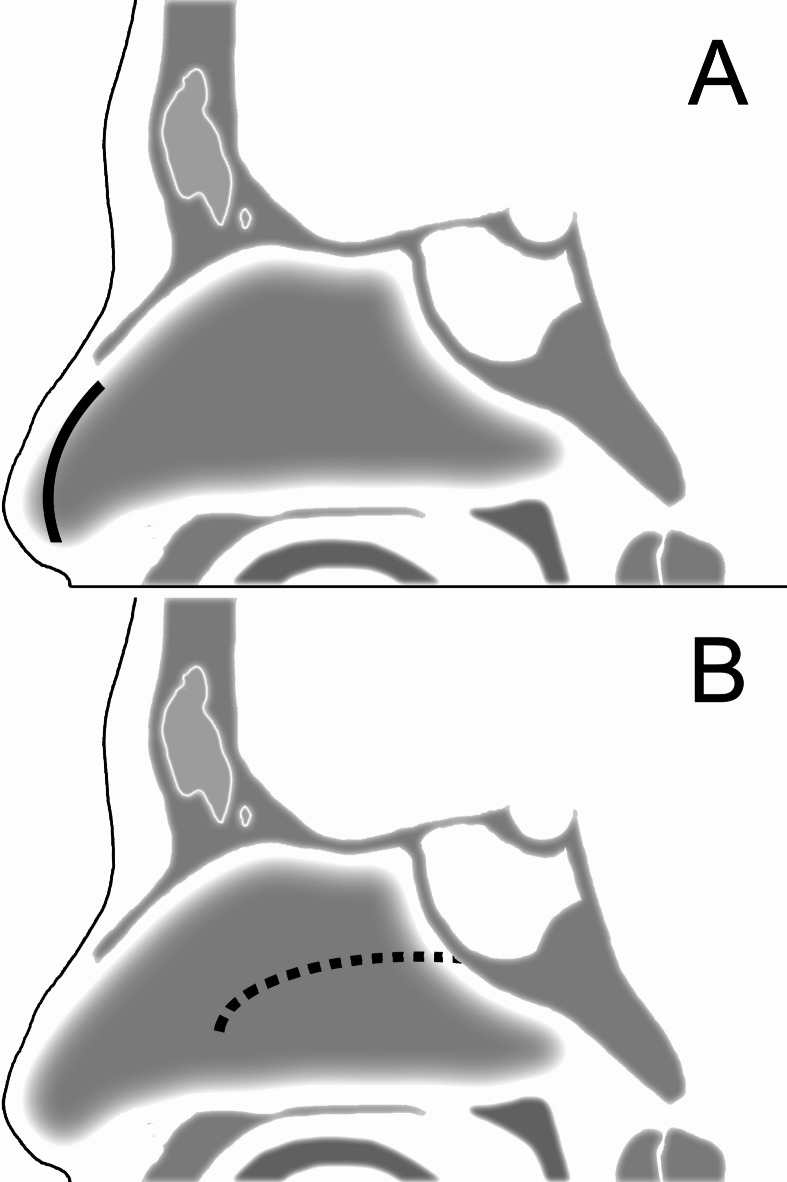
(**A**) A nasal septal mucosal incision known as the Killian incision (black line). The Killian incision is a vertical mucosal incision just posterior to the mucocutaneus transition site on the nasal septal mucosa. (**B**) A nasal septal mucosal incision known as the rescue flap incision (black dotted line). For preservation of both the olfactory mucosa and the posterior septal branch of the sphenopalatine artery, this incision is initiated from the sphenoid ostium and it goes straight back to the anterior margin of the middle turbinate. The incision line should be almost parallel to the hard plate line not to injure the posterior septal branch of the sphenopalatine artery.

For the Killian incision, a vertical mucosal incision was made just posterior to the mucocutaneus transition site on the nasal septal mucosa (Fig. 3A). The submucoperiosteal semi-sharp dissection was conducted using a Suction curette (Medtronic, Minneapolis, MN, USA) to expose the vomer bone and sphenoid rostrum. In cases with nasal septal deviation, a septoplasty was performed to improve the maneuverability of the instruments and facilitate the following procedures. At the site of the Killian incision, the incised mucosal edge was sutured on the inner mucosal surface of the alar nasalis to secure the entrance of the created submucosal tunnel by the Killian incision; this in turn enabled the surgical instruments to be utilized through this incision^16^. At the boundary between the cartilaginous and bony septum, a transseptal incision was made; thereafter, contralateral submucosal dissection to the sphenoid rostrum was conducted and the posterior part of the nasal septum was removed. Next, the rescue flap incision, which is a linear incision parallel to the hard plate, was made on the contralateral nasal septal mucosa with a thin-tip monopolar cautery from the point of the inferior edge of the sphenoid ostium to the depth of the anterior edge of the middle turbinate (Fig. 3B)^15^. At this mucosal incision, care was taken not to injure the posterior septal branches of the sphenopalatine artery and to preserve the olfactory mucosa. This rescue flap incision could be extended laterally from the sphenoid ostium to the inferior edge of the superior turbinate by cutting into the inferior part of the superior turbinate, allowing the anterior sphenoidotomy to expand laterally. After making the rescue flap incision, the superior edge of the incised mucosal flap was adjusted inferolaterally to ensure that it did not hinder the following procedures. As a result of the mucosal incisions, the nasal septal mucosa on the side of the Killian incision was almost completely preserved. Concurrently, on the side of the rescue flap incision, the mucosa on the septal cartilage was not dissected and remained attached to the septal cartilage to prevent necrosis of the cartilage. Thereafter, the binostril bimanual procedure could be conducted. The lower third of the superior turbinate was generally resected in cases of a large posterior ethmoid sinus, also known as the Onodi cell; however, the middle turbinate was preserved. Following a wide anterior sphenoidotomy and septectomy in the sphenoid sinus which preserved the sphenoid sinus mucosa to be used at reconstruction, the sellar floor and dura were opened, and the tumor was resected. After tumor resection, dural plasty using the suturing technique was performed in cases with an intraoperative cerebrospinal fluid (CSF) leak. Then, the preserved and pedicled sphenoid sinus mucosal flap was placed on the sellar dura in cases of an absent or a low-flow intraoperative CSF leak. Conversely, NSF was harvested and used in cases of an intraoperative high-flow CSF leak. Details of the reconstruction technique have been previously reported^19^.

Even in recurrent cases that initially underwent the PTSA with K-R incision, this technique could be repeated (Supplementary Video 2).

### Statistical analysis

All statistical analyses were performed using IBM SPSS 24.0 (IBM, Chicago, Illinois, USA) with Mann–Whitney U and Fisher exact tests. P-values less than 0.05 were considered statistically significant.

### Ethics

The study was approved by the Ethics Review Committee of the University of Tsukuba Hospital (R1-231) and conducted in accordance with the Declaration of Helsinki. Due to the retrospective nature of the study, the Ethics Review Committee of the University of Tsukuba Hospital waived the need of obtaining informed consent.

## Results

### Patient background

The clinical and demographic characteristics and surgical results for the patients underwent EEA using PTSA with K-R incision and direct approach are shown in Table 1. In the 10 patients with recurrent tumor, although they had undergone a prior endonasal surgery using PTSA, PTSA with K-R incision was successful in the present surgery. Four and six of the 10 patients had undergone prior surgery in other hospitals and our hospital, respectively. The median neurosurgical follow-up period for the 189 patients using PTSA with K-R incision was 58 months (range; 1–101) and 6 patients using direct approach was 77.5 months (range; 66–89).

**Table 1:** Summary of clinical and radiological characteristics, surgical outcomes, and postoperative rhinological follow-up in patients who underwent the para/transseptal approach using a combination of the Killian and contralateral rescue flap incision techniques and direct endonasal approach for PitNET.

Clinical and radiological characteristics	PTSA with K-R incision (N = 189)	Direct endonasal approach (N = 6)
Neurosurgical follow-up period (median, range)	58 (1–101) months	77.5 (66–89) months
Age (average ± STDV)	56.3 ± 17.2	56.0 ± 17.6
Sex	Female 104 (55.0%), Male 85 (45.0%)	Female 2 (33.3%), Male 4 (66.7%)
Recurrent case (N)	10 (5.3%)	5 (83.3%)
Tumor type	Non-functioning tumor 133 (70.4%), Functioning tumor 56 (29.6%)	Non-functioning tumor 4 (66.7%), Functioning tumor 2 (33.3%)
Maximum tumor diameter (mm; average, range)	26.6 (5–73)	29.5 (9–64)
Knosp grading	Grade 0–1: 48 (25.4%), Grade 2: 51 (27.0%), Grade 3: 61 (32.3%), Grade 4: 29 (15.3%)	Grade 0–1: 3 (50%), Grade 2: 0, Grade 3: 2 (33.3%), Grade 4: 1 (16.7%)
Bilateral Knosp grade 3 or 4	10 (5.3%)	2 (33.3%)
Side of lateral tumor extension	Right: 58 (30.7%), Left: 61 (43.3%), Bilateral: 21 (11.1%), None: 49 (25.9%)	Right: 1 (16.7%), Left: 1 (16.7%), Bilateral: 1 (16.7%), None: 3 (50.0%)
Side of septum deviation	Right: 48 (43.4%), Left: 58 (30.7%), None: 83 (43.9%)	Right: 2 (33.3%), Left: 0, None: 4 (66.7%)
Discrepancy of the side between lateral tumor extension and septum deviation	31 (26.1%) [Knosp2: 9/36 (25.0%), Knosp3: 15/56 (26.8%), Knosp4: 7/27 (25.9%)] (n = 119)*	1 (50.0%) [Knosp3: 1/2 (50.0%))] (n = 2)*
Surgical outcome	(N = 189)	(N = 6)
Extent of tumor resection	GTR 146 (77.2%), STR 38 (20.1%), PR 5 (2.6%)	GTR 3 (50.0%), STR 1 (16.7%), PR 2 (33.3%)
Achieving GTR associated with Knosp grading	Knosp 0–2: 90 (90.9%), Knosp 3–4: 56 (62.2%)	Knosp 0–2: 3 (100%), Knosp 3–4: 0 (0%)
Harvesting NSF	5 (2.6%)	1 (16.7%)
CSF leakage	3 (1.6%)	0 (0%)
Postoperative rhinological follow-up	(N = 168)**	(N = 5)**
Follow-up period (median, range)	6 (3–32) months	6 (5–9) months
Posterior septal perforation	18 (10.7%)	5 (100%), large septal perforation in all the cases
Anterior septal perforation	6 (3.6%)	0
Anterior septal perforation requiring surgical intervention	1 (0.6%)	0
Epistaxis requiring hemostatic procedures	6 (3.6%)	0
Sinusitis requiring treatments	Antibiotics only: 31 (18.5%), Surgical intervention: 0	0
New paranasal mucocele	0	0

Abbreviations: PTSA with K-R incision, para/transseptal approach using the combination of a Killian and a contralateral rescue flap incision; STDV, standard deviation; NSF, nasal septal flap; CSF, cerebrospinal fluid; GTR, gross total resection; STR, subtotal resection; PR, partial resection.

*Patients with lateral tumor extension to one side.

**Patients with available rhinological follow-up data, excluding patients for whom NSF was made.

### The selection of the side of the rescue flap incision based on the tumor lateral extention

Overall, 31 (26.1%) of 119 patients with Knosp 2–4 had a discrepancy between the side of the lateral tumor extension and the nasal septum deviation. Among 25 of the 31 patients, the side of the rescue flap incision was selected as the side of the tumor with lateral extension. In the remaining six patients, the side was selected as the septum deviation. Regarding the extent of tumor resection, GTR was achieved in 146 (77.2%) patients. A NSF was harvested in five (2.6%) patients on the side with the Killian incision after tumor removal because of an unexpected intraoperative high-flow CSF leakage.

### Postoperative nasal observation

In total, 168 and 5 patients using PTSA with K-R incision and direct approach, respectively, had available data on otolaryngological fiberscopic findings during the postoperative follow-up; this excluded the six cases for whom NSF was used. A anterior and posterior septal perforation was observed in six (3.6%) and 18 (10.7%) of the 169 patients using PTSA with K-R incision, respectively (Fig. 2B, left and center) Therefore, in the majority of patients using PTSA with K-R incision, the nasal septal mucosa was completely preserved on the Killian incision side and was mostly preserved on the contralateral side of the rescue flap incision, with the exception of the area around the sphenoid ostium (Fig. 2A). On the other hand, in the 5 patients using the direct approach, large posterior septal perforation was observed in all cases (Fig. 2B, right). In all the patients using PTSA with K-R incision, nasal septal deviation was resolved based on the direct observation by the otolaryngologist and on the CT findings. Postoperative nasal symptoms were evaluated by telephone in all six patients with an anterior septal perforation and in 16 of 19 patients with a posterior septal perforation. Of the six patients with anterior septal perforation, three were symptomatic. In one of the three symptomatic patients, the symptoms were so severe that surgical intervention was required. Conversely, only two of the 16 patients with posterior septal perforation were symptomatic. One of the six patients using the direct approach was excluded because it would have been challenging to account for the effects of multiple surgeries for recurrences and the proton therapy which was conducted as adjuvant therapy for aggressive PitNET. Four of the remaining five patients were interviewed by telephone. One of the four patients complained of an increased crusting after the surgery. Postoperative epistaxis occurred in six (3.6%) of the 168 and no patients using PTSA with K-R incision and direct approach, respectively. Sinusitis requiring antibiotic therapy occurred in 31 patients using PTSA with K-R incision, but none required surgery.

In patients using PTSA with K-R incision, having a history of previous pituitary surgery was a significantly associated factor for postoperative posterior septal perforation (p = 0.041) (Table 2).

**Table 2:** Factors associated with anterior or posterior septal perforation.

	Anterior septal perforation, present (N = 6)	Anterior septal perforation, absent (N = 162)	P value	Posterior septal perforation, present (N = 18)	Posterior septal perforation, absent (N = 150)	P value
Sex (female)	5 (83.3%)	88 (54.3%)	0.227	7 (38.9%)	86 (57.3%)	0.209
Age (average ± STDV)	57.8 ± 11.3	55.4 ± 17.1	0.728	57.4 ± 14.8	55.2 ± 17.2	0.601
Knosp grade 3 or 4	4 (66.7%)	75 (46.3%)	0.422	7 (38.9%)	72 (48.0%)	0.618
Maximum tumor diameter (mm; average ± STDV)	28.2 ± 6.5	26.0 ± 10.5	0.615	27.2 ± 8.2	25.9 ± 10.6	0.635
Achieving gross total resection	4 (66.7%)	128 (79.0%)	0.610	17 (94.4%)	115 (76.7%)	0.126
Existence of nasal septum deviation	3 (50.0%)	94 (58.0%)	0.698	13 (72.2%)	84 (56.0%)	0.216
Discrepancy of the side between lateral tumor extension and nasal septum deviation	0	31 (19.1%)	0.594	4 (22.2%)	27 (18.0%)	0.747
Degree of septal deviation (mm; average ± STDV)	2.1 ± 2.5	2.9 ± 2.8	0.520	3.2 ± 2.5	2.9 ± 2.9	0.591
History of previous pituitary surgery	0	8 (5.0%)	1.000	3 (16.6%)	5 (3.4%)	0.041
Acromegaly	0	34 (21.0%)	0.350	3 (16.7%)	31 (20.7%)	1.000

P-values less than 0.05 were considered statistically significant.

## Discussion

Our method using PTSA with K-R incision in EEA for PitNETs enables the preservation of septal mucosa completely on the side of Killian incision, as well as to expand anterior sphenoidotomy laterally on the side of the rescue flap incision. Our study is novel for two reasons. Firstly, our strategy was to select the side of the rescue flap incision as the same side of the lateral tumor extension to facilitate tumor resection. Secondly, we assessed postoperative nasal septal perforation by regular rhinological observation with fiberscope in a large number of patients.

Several studies have shown surgical outcomes in EEA for PitNETs using various types of nasal mucosal treatment (Table 3)^4,5,13,14,20,21^. Using the direct approach, a large posterior nasal septum perforation is inevitable and unexpected injury of nasal structures, especially a nasal turbinate, can occur^10,16,20^. A large posterior septal perforation potentially worsens the early postoperative quality of life (QOL), producing a flow reduction into the nose^22,23^. Hong et al. compared olfactory outcomes evaluated using nasal QOL scoring between patients who underwent either the para/transseptal and the direct approach. They found that PTSA could preserve almost all nasal mucosa including the nasal turbinates on one side; the early postoperative olfaction-related nasal QOL was better preserved in patients who underwent PTSA^20^. Furthermore, they reported no difference in operative time between PTSA and direct approach.^20^. In our study, we could not compare the operative time using the PTSA with K-R incision with one using the direct approach, because of the limited number of the patients with using the direct approach. The incidence of postoperative septal perforation has been reported to be 0–4% in EEA^4,5,13,14,20,21^. However, a large posterior septal perforation is inevitable in patients who underwent the direct approach^9,20^, because bilateral posterior parts of septal mucosa are incised in this approach as shown in the Fig. 2B, right. Therefore, a posterior perforation seemed to be ignored and only an anterior septal perforation was counted as a septal perforation in these reports. As shown in the Table 3, in the three previous reports of patients underwent direct approach, a septal perforation was not assessed in the 2 reports. And in the remaining 1 report, no patient had a septal perforation, that means they counted only an anterior septal perforation as a septal perforation. The reason why they ignored a posterior septal perforation is unclear, but we expect that only an anterior septal perforation is symptomatic in most cases. In the present study, the frequency of an anterior septal perforation was only 6 (3.6%), which is comparable to the results of other reports. In addition, the rate and category of septal perforations were accurately evaluated in our study because only the patients underwent direct observation by otolaryngologists were included. On the other hand, in other literatures, it is unclear that if all the patients underwent direct observation by otolaryngologist, if both anterior and posterior septal perforation were assessed as a septal perforation, or if only a symptomatic case was counted as having a septal perforation. Only one report by Hong et al., they used the same technique as one in our study, clarified that definition of a septal perforation, which was an anterior septal perforation, and they had no patients with septal perforation^20^. In their report, they described that the otolaryngologist assessed at 1, 3, and 6 months after surgery, however, it was unclear if they directly observed all the patients with fiberscope. As previously reported, anterior septal perforation reduces the amount of air flow into the nasal cavity and is more problematic^23,24^. We also found that patients with an anterior septal perforation had more frequent nasal complaints than those with a posterior septal perforation. Although there is a risk of anterior septal perforation with PTSA, the incidence in our study was low (3.6%), and only one patient (0.6%) underwent revision surgery to close the perforation. The treatment by the collaborative team of otolaryngologist and neurosurgeons may have contributed to this outcome.

**Table 3:** Comparison of surgical outcomes among patients who received endoscopic endonasal surgeries for pituitary endocrine tumor.

Authors	Number of cases	Approach	GTR	GTR in Knosp 3 and 4	Septal perforation	Regular observation by rhinologist to detect septal perforation	CSF leakage	Epistaxis
Cardinal et.al	404	Binostril direct	208 (58.0%)	29/99 (28.0%)	NR	NR	16 (4.0%)	17 (4.0%)
Paluzzi et.al	555	Binostril direct	359 (65.3%)	42/156 (26.9%)	0	Yes	28 (5.0%)	6 (1.0%)
Gondim et.al	228	Binostril direct	181 (79.3%)	NR	NR	NR	8 (3.1%)	7 (2.7%)
Hong et.al	51	Binostril para/transseptal with K-R	37 (72.5%)	NR	0*	Yes	0	NR
Fujimoto et.al	91	Binostril para/transseptal with K-R	NR	NR	4 (4.4%)	Yes	4 (4.4%)	3 (3.3%)
Han S. et.al	200	Uninostril para/transseptal	174 (87.0%)	0/14 (0%)	NR	NR	7 (3.5%)	0 (0%)
Present study	189	Binostril para/transseptal with K-R	146 (77.2%)	56/90 (62.2%)	Posterior 18 (10.7%)** Anterior: 6 (3.6%)** Surgically treated: 1 (0.6%)**	Yes	3 (1.6%)	6 (3.6%)

Abbreviations: NR, not reported; CSF, cerebrospinal fluid; GTR, gross total resection.

*Only anterior septal perforation is reported. **Total = 168 patients with available follow-up data.

In some previous reports of surgical methods for PitNETs using the direct approach, a middle turbinectomy is performed, which creates an obstacle when inserting surgical instruments from outside the nasal cavity^25,26^. Because the middle turbinate is necessary to maintain nasal function, middle turbinectomy can cause nasal obstruction and epistaxis in postoperative patients^27,28^. In our method, sacrifice of the middle turbinate is unnecessary because a surgical corridor is created on the median side of the nasal cavity. Our method of preserving the middle turbinate may contribute to improvement of the patient’s nasal QOL after surgery.

Recently, various modifications to the methods first reported by Stamm et al.^10^ have been reported^4,11,16,20^. These methods ensure that the unilateral nasal septal mucosa is kept intact, and they allow for anterior sphenoidotomy to be laterally expanded on the other side. The major difference between the present and past studies^4,11,16,20^ is that we selected the rescue flap incision side as that used for the lateral tumor extension. In the previous studies, a Killian incision was made on the determined side based on the surgeon’s preference^4,11,20^. Considering maneuverability of instruments during tumor resection, lateral widening of the anterior sphenoidotomy is crucial, especially in cases of significant lateral tumor extension (cases with Knosp 3 or 4). For the lateral widening of the anterior sphenoidotomy, the rescue flap incision is superior to the Killian incision because preserved nasal septal mucosa hinders lateral widening. Bilateral widening of the anterior sphenoidotomy is rarely required because a normal pituitary gland medial to the cavernous sinus wall on one side may prevent tumor extension into the cavernous sinus in most cases^29^. In our study, there were only 10 (5.3%) cases of bilateral Knosp 3 or 4. The resection in our study was comparable to that reported in previous studies using the direct approach^13,14,21^. Furthermore, among cases of Knosp 3 or 4, the GTR rate was significantly higher than that obtained using the direct approach (Table 3). Although our superior GTR rate is possibly due to the method used for tumor resection, based on our findings, the current method was not inferior to the direct approach regarding the extent of resection. However, previous reports using the PTSA have not described the GTR rate specifically among cases with Knosp 3 or 4, therefore, the overall GTR rate for EEA using the PTSA remains unclear.

Selection of the side for mucosal incision in a septoplasty is controversial. Some prefer that the side of incision is the same as the concave side of the deviated septum^30^; others prefer the right or left side in all cases irrespective of the side of the septal deviation^31,32^. Our otolaryngologist team prefers the former strategy of selection because the septal mucosa is thick and it is easy to preserve on the concave side of the deviated septum. In our study, the side of septum deviation and lateral tumor extension was matched in the majority of patients; therefore, paraseptal submucosal dissection was easy in most cases. The reason for this is unclear because no other reports have evaluated these findings; thus, further studies are required.

This study was subject to several limitations. First, this was a retrospective analysis conducted in a single institution. Second, the majority of patients underwent PTSA with K-R incision and the lack of the sufficient control group, therefore the superiority of our method over the direct approach for nasal septal mucosa preservation cannot be accurately evaluated. Third, we did not evaluate the patient’s QOL; therefore, the association of septal perforation and patient’s QOL was not determined.

## Conclusion

PTSA with K-R incision is a useful method for preserving the nasal septal mucosa on one side and gaining a sufficient surgical corridor with lateral expansion of the anterior sphenoidotomy on the other side in EEA for PitNETs. Our strategy considers the lateral tumor extension side as the side for nasal septal mucosa incision; this may improve maneuverability of instruments during tumor resection and contribute to achievement of preferable resection rates for PitNETs with lateral extension.

## Electronic supplementary material

Below is the link to the electronic supplementary material.

Supplementary Material 1

Supplementary Material 2

Supplementary Material 3

## Data Availability

The datasets generated and analyzed during the current study are not publicly available due to contains a lot of personal identification information but are available from the corresponding author on reasonable request. The data set is kept on a PC in a locked locker in the Department of Neurosurgery at the University of Tsukuba.

## Abbreviations

EEA: Endoscopic endonasal approach
PTSA: Para/transseptal approach
NSF: Nasal septal flap
MRI: Magnetic resonance imaging
CT: Computed tomography
GTR: Gross total resection
STR: Subtotal resection
PR: Partial resection
CSF: Cerebrospinal fluid
QOL: Quality of life
