# Supplementary material for: Endoscopic endonasal approach for pituitary neuroendocrine tumor with septal mucosa incision tailored to tumor extension intending unilateral septal mucosa preservation
Source: Sci Rep. 2025 Jan 9;15:1489. doi: 10.1038/s41598-024-84334-y (PMC11718052; doi:10.1038/s41598-024-84334-y)
Supplement: Supplementary file 3 — Supplementary Material 3 [file 41598_2024_84334_MOESM3_ESM.docx]

Video 1. Surgical technique of PTSA with K-R incision

Surgical technique of para/transseptal approach with combination of Killian incision and contralateral rescue flap incision (PTSA with K-R incision) are presented.

Video 2. Illustrated case underwent PTSA with K-R incision

Illustrated case of non-functioning pituitary neuroendocrine tumor with lateral tumor extension underwent a para/transseptal approach with combination of a Killian incision (PTSA with K-R incision) and contralateral rescue-flap incision are presented. Furthermore, in recurrent cases that initially underwent the PTSA with K-R incision, this technique could be repeated, which was also presented.
